# Hepatocyte- Specific Deletion of ARNT (Aryl Hydrocarbon Receptor Nuclear Translocator) Results in Altered Fibrotic Gene Expression in the Thioacetamide Model of Liver Injury

**DOI:** 10.1371/journal.pone.0121650

**Published:** 2015-03-26

**Authors:** Christopher Scott, Kuan Cha, Renuka Rao, Christopher Liddle, Jacob George, Jenny E. Gunton

**Affiliations:** 1 Diabetes, Obesity & Endocrinology Group, Westmead Millennium Institute, Westmead Hospital, Sydney, NSW, Australia; 2 Faculty of Medicine, University of Sydney, Westmead, Sydney, NSW, Australia; 3 Diabetes and Transcription Factors Group, Garvan Institute of Medical Research, Sydney, NSW, Australia; 4 The Storr Liver Unit, Westmead Millennium Institute and University of Sydney, Westmead Hospital, Sydney, NSW, Australia; 5 St. Vincent’s Clinical School, University of NSW, Sydney, NSW, Australia; 6 Department of Diabetes and Endocrinology, Westmead Hospital, Sydney, NSW Australia; University of Dundee, UNITED KINGDOM

## Abstract

**Background & Aims:**

Recent studies have shown that increased expression of liver hypoxia inducible factor 2-α (HIF-2α) leads to liver inflammation and a pro-fibrotic gene expression signature. Aryl hydrocarbon Receptor Nuclear Translocator (ARNT) is required for HIF-2α transcriptional activity and has previously been shown to regulate hepatic metabolism in mice. In these studies we examined the role of hepatocyte ARNT in the thioacetamide (TAA)-induced model of liver fibrosis.

**Methods:**

Hepatocyte-specific *ARNT*-null (LARNT) mice were created using an albumin promoter-driven Cre recombinase. LARNT and floxed control (FC) littermates were placed on chow diet and received twice weekly intraperitoneal injections of 0.15mg/g body weight of TAA for 13 weeks.

**Results:**

TAA treated LARNT and FC mice had a similar pattern of fibrosis. Quantification of Sirius red histology staining and hydroxyproline content revealed mixed results in terms of collagen deposition in LARNT livers. There was no significant difference in hepatocyte apoptosis or proliferation, as assessed by cleaved Caspase-3 and Ki67 respectively. LARNT mice had decreased macrophage accumulation, and decreased liver mRNA expression of *Col1A1*, *Col1A2*, *Col5A1*, *Tgfβ1*, *Tgfβ2*, *Timp1* and *Timp2*.

**Conclusions:**

Deletion of hepatocyte ARNT leads to altered expression of collagen associated mRNA and reduced macrophage infiltration in the TAA-induced model of liver fibrosis. It appears that hepatocyte ARNT is not a requirement for initiation of liver fibrogenesis, but does regulate pro-fibrotic gene expression and macrophage accumulation.

## Introduction

Liver fibrosis represents the result of an unsuccessful wound repair processes characterised by the accumulation of extracellular matrix (ECM) proteins including pathological collagens. It is a feature of the majority of chronic liver diseases including non-alcoholic steatohepatitis (NASH), chronic viral hepatitis and alcohol abuse [1]. In some patients liver fibrosis leads to liver cirrhosis with portal hypertension, hepatocellular dysfunction and increased risk of hepatocellular carcinoma [2,3]. Hepatic stellate cells, which assume an activated phenotype in response to liver injury and inflammation, are the major source of pathological matrix components [1,3].

The Aryl hydrocarbon Receptor Nuclear Translocator (ARNT) acts as a general partner for members of the bHLH/PAS family of transcription factors. It heterodimerises with other bHLH/PAS family members including Hypoxia-Inducible Factor-1-α (HIF-1α), Hypoxia-Inducible Factor-2-α (HIF-2α) and Aryl hydrocarbon Receptor (AhR) to form active transcription complexes which regulate genes involved in hypoxic-responses, cell survival, proliferation, glycolysis, angiogenesis and response to xenobiotics [4,5,6,7].

Previous research has indicated a role for these transcription factors in both liver function and fibrosis [8,9,10]. It has been shown using a model of acute hepatocyte-specific double deletion of vHL and HIF-1α or HIF-2α under control of the Cre-ER system, that acute elevations in hepatic HIF-2α resulted in increased liver inflammation and pro-fibrotic mRNA expression [9]. In addition, AhR knockout mice exhibit a liver phenotype characterised by transient steatosis, increased hepatocyte apoptosis and fibrosis driven by increased TGF-β1 expression [11,12,13]. Another study suggested that signalling through AhR may sensitise hepatocytes to FAS induced apoptosis [14]. The effects of hepatocyte HIF-1α deletion have varied in different studies. For example, *Hif-1α* deletion in mice has been shown to be protective against fibrosis in a bile duct ligation model but there are conflicting results in a model of ethanol-induced fatty liver [15,16,17].

In the present study hepatocyte specific *ARNT*-null (LARNT) mice were created to investigate the role of hepatocyte ARNT in liver fibrosis. There was reduced hepatic macrophage infiltration and decreased mRNA expression of *Col1A1*, *Col1A2*, *Col5A1*, *Tgfϐ1*, *Tgfϐ2*, *Timp1* and *Timp2*. However, the histological pattern of fibrosis was equivalent. This suggests that hepatocyte ARNT is not required for initiation of fibrosis.

## Materials and Methods

### Animal studies

Floxed ARNT mice (Kindly provided by Frank J. Gonzalez) were created as previously described [4,18,19]. Hepatocyte-specific ARNT null (LARNT) mice were created by breeding these mice with Albumin-Cre mice (kindly provided by David James, Garvan Institute) to produce LARNT and floxed-control (FC) offspring. All mice were on an inbred C57Bl/6 background. All procedures were approved by the Garvan Animal Ethics Committee.

For basal histology 20 week old male chow fed mice were culled and livers resected for histology (N = 6 LARNT and 7 FC mice). For TAA studies, 10 week old male chow fed mice were injected IP twice weekly with 0.15mg/g TAA dissolved in sterile water for 13 weeks (N = 7 per genotype). Livers were then divided for formalin fixation and snap-freezing in liquid nitrogen for gene expression. All animals were culled after an overnight fast (16 hours).

### Gene expression analysis

Livers from LARNT and FC mice were homogenized in RLT buffer (Qiagen, Valencia, CA). RNA was isolated and cDNA was synthesized as previously described. Real-time PCR was performed using specific primers and Sybr Green PCR master mix (Applied Biosystems, Melbourne Australia), and amplification was performed in an ABI7900 light-cycler (Applied Biosystems). Results were normalised to *18S* ribosomal RNA, which did not differ between groups (data not shown). Primer sequences are shown in **Table 1**. Statistical analysis was performed on corrected CT value, fold change of mRNA expression was calculated and graphed using the 2^∆∆CT^ method.

**Table 1:** RTPCR primer list.

**mRNA**	**Fwd primer**	**Reverse Primer**
*18s*	ggtgcatggccgttctta	tgccagagtctcgttcgtta
*Arnt*	tctccctcccagatgatgac	caatgttgtgtcgggagatg
*Bak1*	cgctacgacacagagttcca	ggtagacgtacagggccaga
*Bax*	tgcagaggatgattgctgac	gatcagctcgggcactttag
*Bcl-2*	tctgaaggattgatggcaga	catcagccacgcctaaaagt
*Bcl-xl*	ccattgctaccaggagaacc	aggagctggtttaggggaaa
*Col1a1*	actgcaacatggagacaggtcaga	atcggtcatgctctctccaaacca
*Col1a2*	aggcgtgaaaggacacagtggtat	tcctgcttgacctggagttccatt
*Col5a1*	agattacgaagttcctcagccgca	atcatccagaatccgggagccaaa
*Col7a1*	tgaggaccctgttgcctctcattt	attggctacttggctcagagagca
*Cxcl-1*	tggctgggattcacctcgaa	tatgacttcggtttgggtgcag
*Cxcl-10*	tggctagtcctaattgcccttggt	tcaggaccatggcttgaccatcat
*F480*	ctttggctatgggcttccagtc	gcaaggaggacagagtttatcgtg
*Il-6*	ccagagatacaaagaaatgatgg	actccagaagaccagaggaaat
*Mcp-1*	tcacctgctgctactcattcacca	tacagcttctttgggacacctgct
*Mmp9*	gaaggcaaaccctgtgtgtt	agagtactgcttgcccagga
*Sma*	gagaagcccagccagtcg	ctcttgctctgggcttca
*Tgfϐ1*	tttggagcctggacacacagtaca	tgtgttggttgtagagggcaagga
*Tgfϐ2*	agtggcttcaccacaaagacagga	attagacggcacgaaggtacagca
*Timp1*	ggtgtgcacagtgtttccctgttt	tccgtccacaaacagtgagtgtca
*Timp2*	acacgcttagcatcacccagaaga	tgatcttgcactcacagcccatct
*Tnfα*	tctcatgcaccaccatcaaggact	tcgaggctccagtgaattcggaaa

### Liver histology

Livers were removed from floxed-control and LARNT mice and the left lobe fixed in 10% buffered formalin. Tissue was paraffin-embedded and 5μm sections were stained with hematoxylin and eosin (H&E) or Sirius Red according to standard protocols. Liver fibrosis in TAA treated animals was scored blinded to genotype using the fibrosis METAVIR score, 0 = no fibrosis, 1 = portal fibrosis without septa, 2 = portal fibrosis with few septa, 3 = numerous septa without cirrhosis and 4 = cirrhosis. Fibrosis content was assessed by quantitating histological collagen staining with ImageJ software in one section per animal, taking care to avoid major vessels and liver capsule. F4/80 staining was performed using the DakoCytomation EnVision+ Dual Link System-HRP (DAB+) Kit (Dako, USA) as per manufacturer’s instructions. After antigen retrieval Rat F480 monoclonal antibody (Abcam, ab6640, USA) was diluted 1:100 in Antibody Diluent (Dako, USA). Caspase 3 antibody (R&D Systems, AF835, USA) was used at a dilution of 1:1000 as described above for F480 staining. Ki67 antibody (Thermoscientific, RM-9106, USA) was used at a dilution of 1:200 as described above. After staining, slides were counterstained using a standard haematoxylin and ethanol dehydration protocol. For apoptosis and proliferation, Caspase 3+ cells and Ki67+ cells were counted in 10 and 9 fields of view respectively at 100x magnification blinded to genotype, and the average result for each section was used in analysis.

### Hydroxyproline assay

Hydroxyproline content was measured in 10mg of liver using a commercial colorimetric assay from Biovision (K555-100) according to manufacturer’s instructions.

### Statistical analysis

Data was analyzed using a 2-tailed unpaired Students t-test using Excel. Mean ±SEM is shown unless otherwise stated. A p value of <0.05 is considered significant.

## Results

### Hepatocyte ARNT deletion does not affect basal liver collagen content

**Fig 1:**
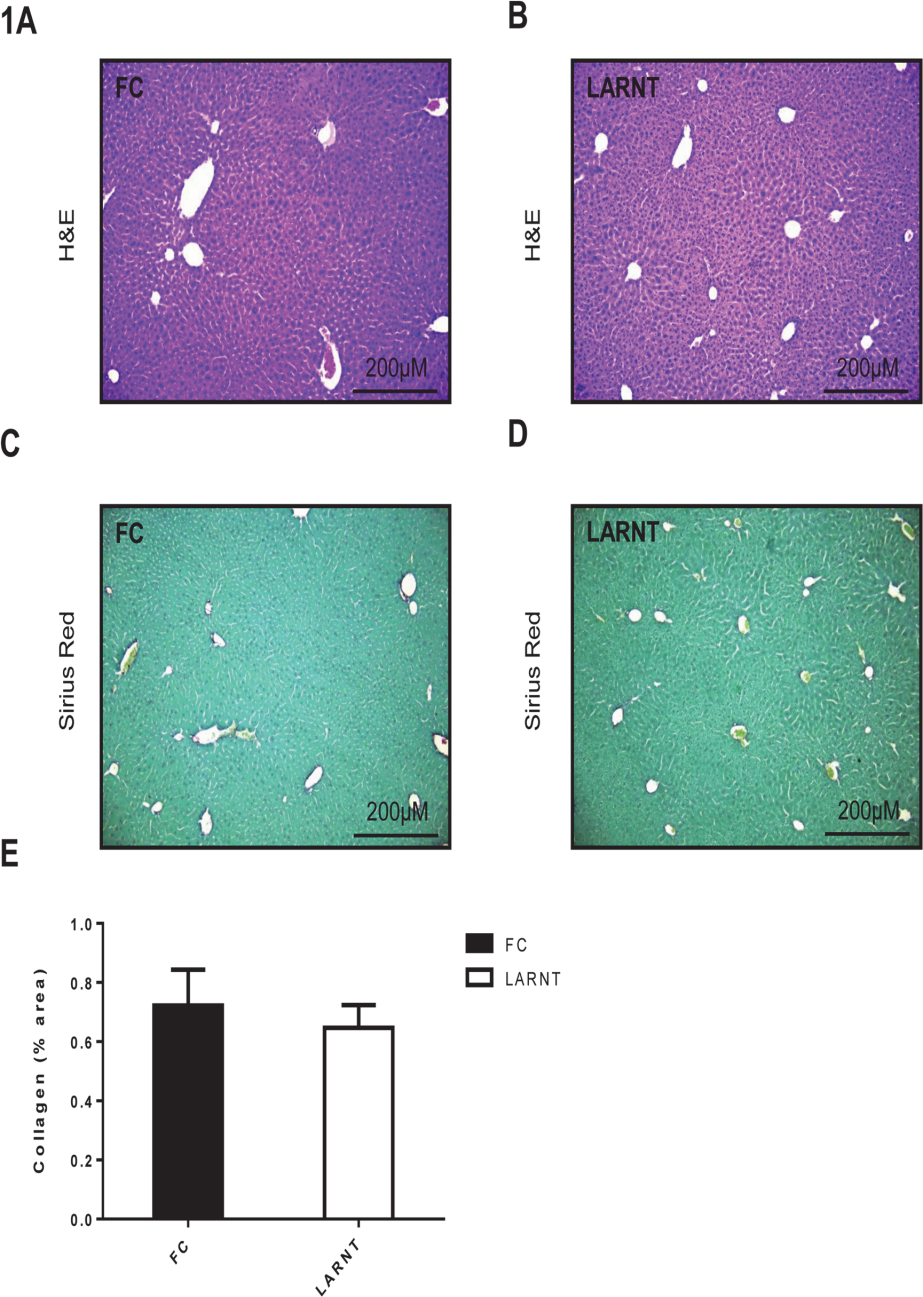
Histology of FC and LARNT livers in chow fed mice. H&E staining under 100x magnification of FC **(A)** and LARNT **(B)** livers. Sirius red staining under 100x magnification of FC **(C)** and LARNT livers **(D)**. Percentage of liver which stained positive for collagen using Image J software in FC (black column) and LARNT (white column) **(E)**. Mean **±**SEM is shown. * = p<0.05. N = 6-7/group.

To assess normal liver histology, H&E staining and Sirius red staining was performed on chow-fed mice. There was no difference observed between FC and LARNT animals in either overall liver morphology or collagen content (**Fig. 1A-D**). Quantification of collagen content in Sirius red stained liver sections using computerised morphometric analysis confirmed there was no difference between LARNT and FC animals (**Fig. 1E**).

### TAA-induced fibrosis is similar in FC and LARNT mice

**Fig 2:**
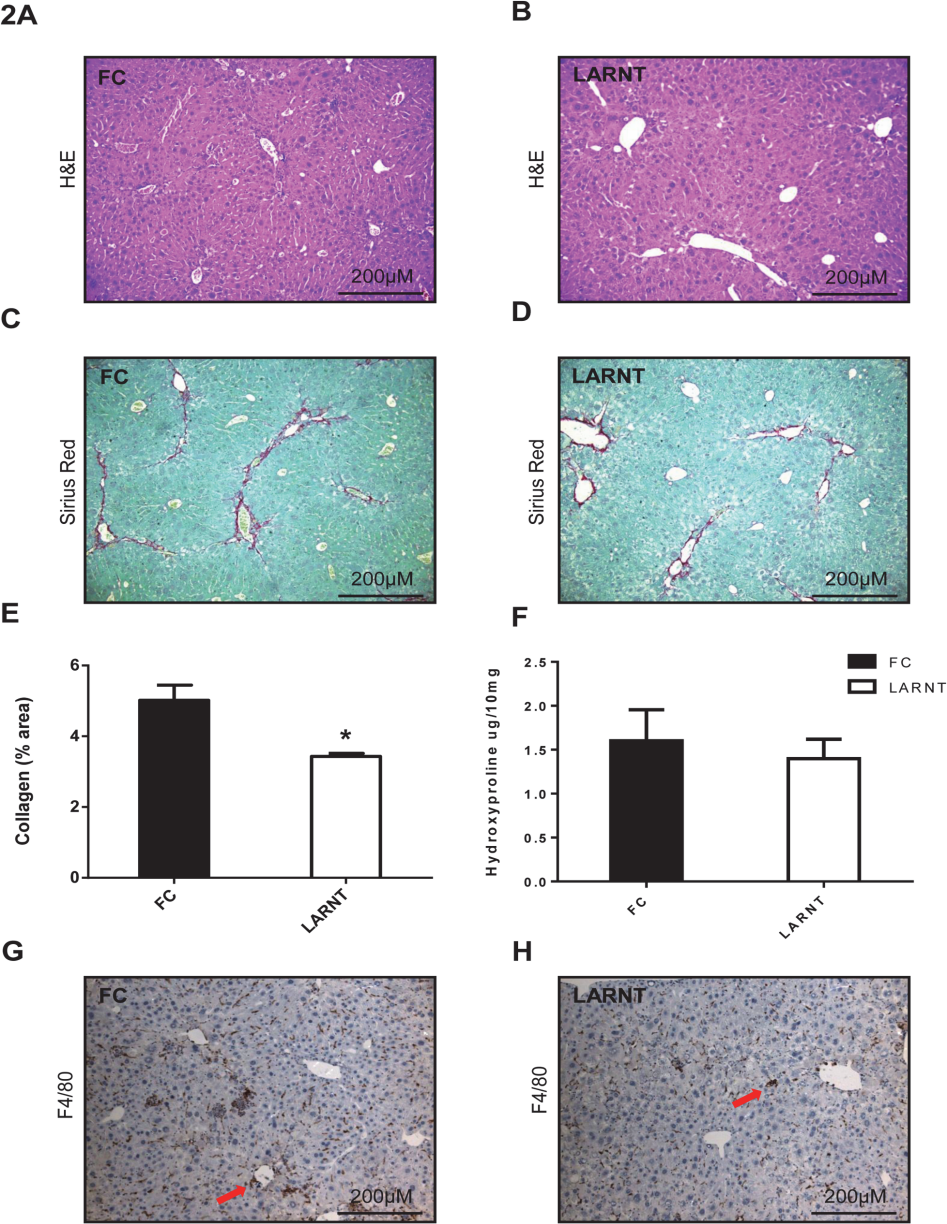
Histology of FC and LARNT livers after the TAA fibrosis model. H&E staining under 100x magnification of FC **(A)** and LARNT **(B)** livers. Sirius red staining under 100x magnification of FC **(C)** and LARNT livers **(D)**. Percentage of liver which stained positive for collagen using Image J software in FC (black column) and LARNT (white column) **(E)**. **(F)** Hydroxyproline assay results from whole liver. F4/80 staining of liver macrophages under 100x magnification of FC **(G)** and LARNT livers **(H).** Mean **±** SEM is shown. * = p<0.05. N = 5-7/group.

H&E staining and Sirius red staining of livers was performed following TAA treatment (**Fig. 2A-D**). Fibrosis, scored blinded to genotype, was similar in both groups (FC 2.0 ±0.4, LARNT 1.6±0.3, p = 0.4). Computerized quantification of Sirius red staining revealed reduced collagen deposition in LARNT mice compared to FC (3.4% of area compared to 5.0%, p = 0.019 **Fig. 2E**). However hydroxyproline assay of whole liver revealed no significant difference in whole liver collagen content, LARNT average 1.4μg/10mg and FC 1.6μg/10mg (**Fig. 2F**, p>0.5). There was reduced macrophage content as assessed by F4/80 expression (**Fig. 2G and H**) in LARNT compared to FC liver.

### Pro-fibrotic gene expression is reduced in LARNT mice

**Fig 3:**
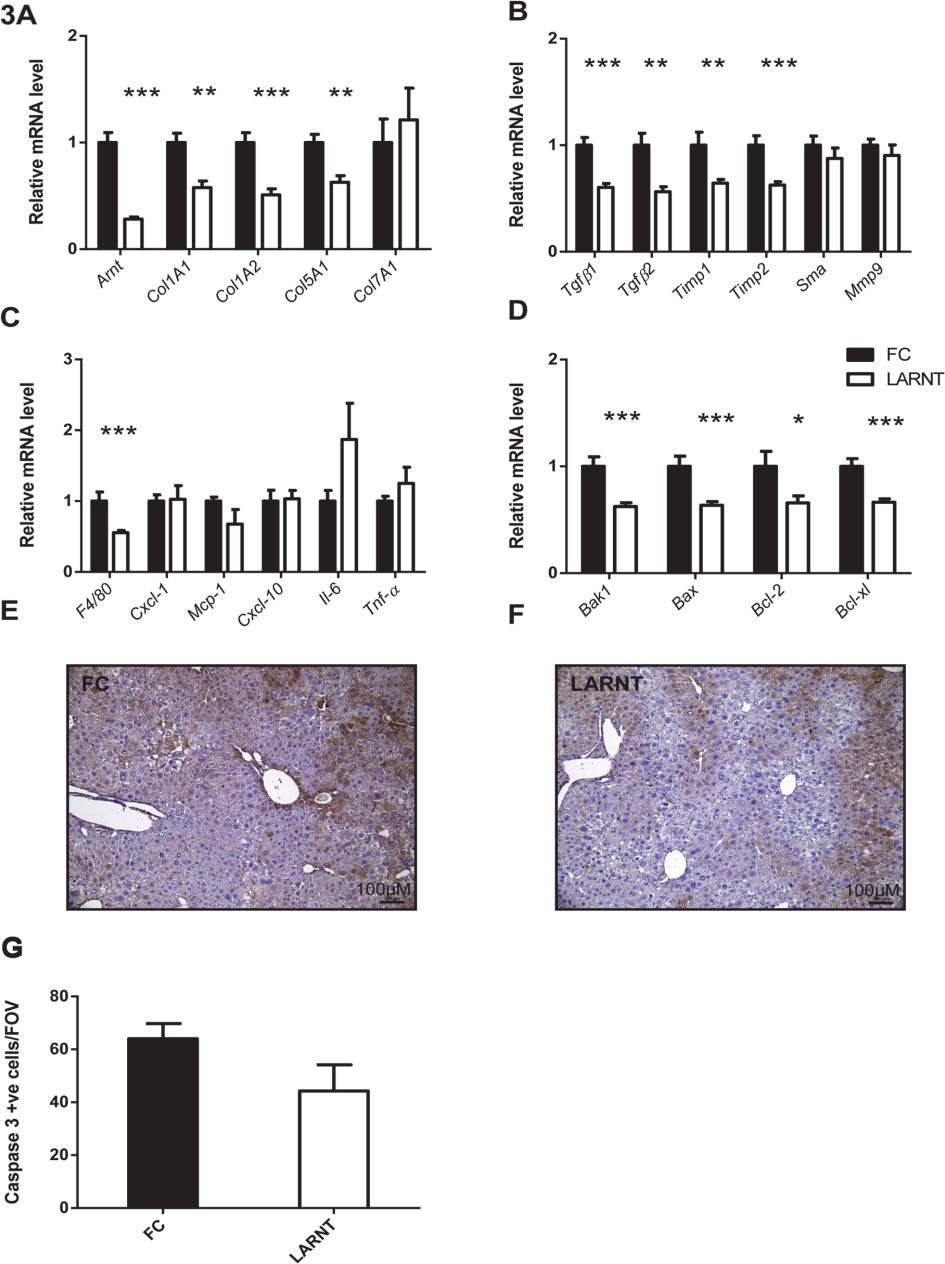
Hepatocyte ARNT deletion alters fibrotic and apoptotic gene expression in the TAA model of fibrosis. Results for FC are shown in black columns and LARNT in white columns. Results are expressed relative to FC level. **(A)** Messenger RNA expression of ARNT and collagen isotypes. **(B)** Expression of genes regulating fibrosis in FC and LARNT mice. **(C)** F4/80 and inflammatory cytokine expression in FC and LARNT mice. **(D)** Apoptotic gene expression in FC and LARNT mice. **(E)** Representative histology of FC liver stained with anti-Caspase 3 at 100X magnification. **(F)** Representative histology of LARNT liver stained with anti-Caspase 3 at 100X magnification. **(G)** Average Caspase 3 positive cell counts per field of view at 100 X magnification. Mean±SEM, * = p<0.05 ** = p<0.01, *** = p<0.001. N = 5-6/group.

As expected, liver *Arnt* mRNA was significantly decreased in LARNT mice (**Fig. 3A**). Significant alterations in fibrosis associated mRNA were found, mRNA for collagen type 1 alpha-1 (*Col1a1*), collagen type 1 alpha-2 (*Col1a2*) and collagen type 5 alpha-1 (*Col5a1*) were all significantly reduced in LARNT livers (**Fig. 3A,** p = 0.0012, 0.00011 and 0.0023 respectively). Additionally, expression of the fibrosis regulating genes, transforming growth factor β1 (*Tgfϐ1*), *Tgfϐ2*, tissue inhibitor of metalloproteinase 1 and 2 (*Timp1* and *2*) were significantly reduced (**Fig. 3B**, 0.000045, 0.0014, 0.0023 and 0.00017 respectively). There was no change in smooth muscle actin (*Sma*), or matrix metallopeptidase 9 (*Mmp-9*) expression (p = 0.29 and 0.21 respectively).

### LARNT mice have reduced liver macrophages but no alteration in cytokine mRNA expression

As expected from immunohistological F4/80 staining, we found a significant decrease in *F4/80* mRNA in LARNT animals (p = 0.0001, **Fig. 3C**). We did not find any significant difference in levels of inflammatory cytokine mRNA expression of chemokine (C-X-C motif) ligand 1 (*Cxcl-1*), monocyte chemotactic protein-1 (*Mcp-1*), C-X-C motif chemokine 10 (*Cxcl-10*), interleukin 6 (*Il-6*) or tumor necrosis factor alpha (*Tnf-α*) (**Fig. 3C** p = 0.53, 0.31, 0.477, 0.93 and 0.79 respectively).

### There were alterations in apoptotic gene expression which did not translate into increased apoptosis

We found reduced expression of apoptotic genes Bcl-2 homologous antagonist killer (*Bak1)*, Bcl-2-associated X protein (*Bax)*, B-cell lymphoma 2 (*Bcl-2)* and B-cell lymphoma-extra large (*Bcl-xl)* (**Fig. 3D,** p = 0.0002, 0.0005, 0.04 and 0.00007 respectively). However this did not translate into a significant alteration in hepatocyte apoptosis at sacrifice, as assessed by cleaved Caspase 3 staining (**Fig. 3E**, **F** and **G**, p = 0.12).

### Proliferation was equivalent in LARNT hepatocytes after TAA

**Fig 4:**
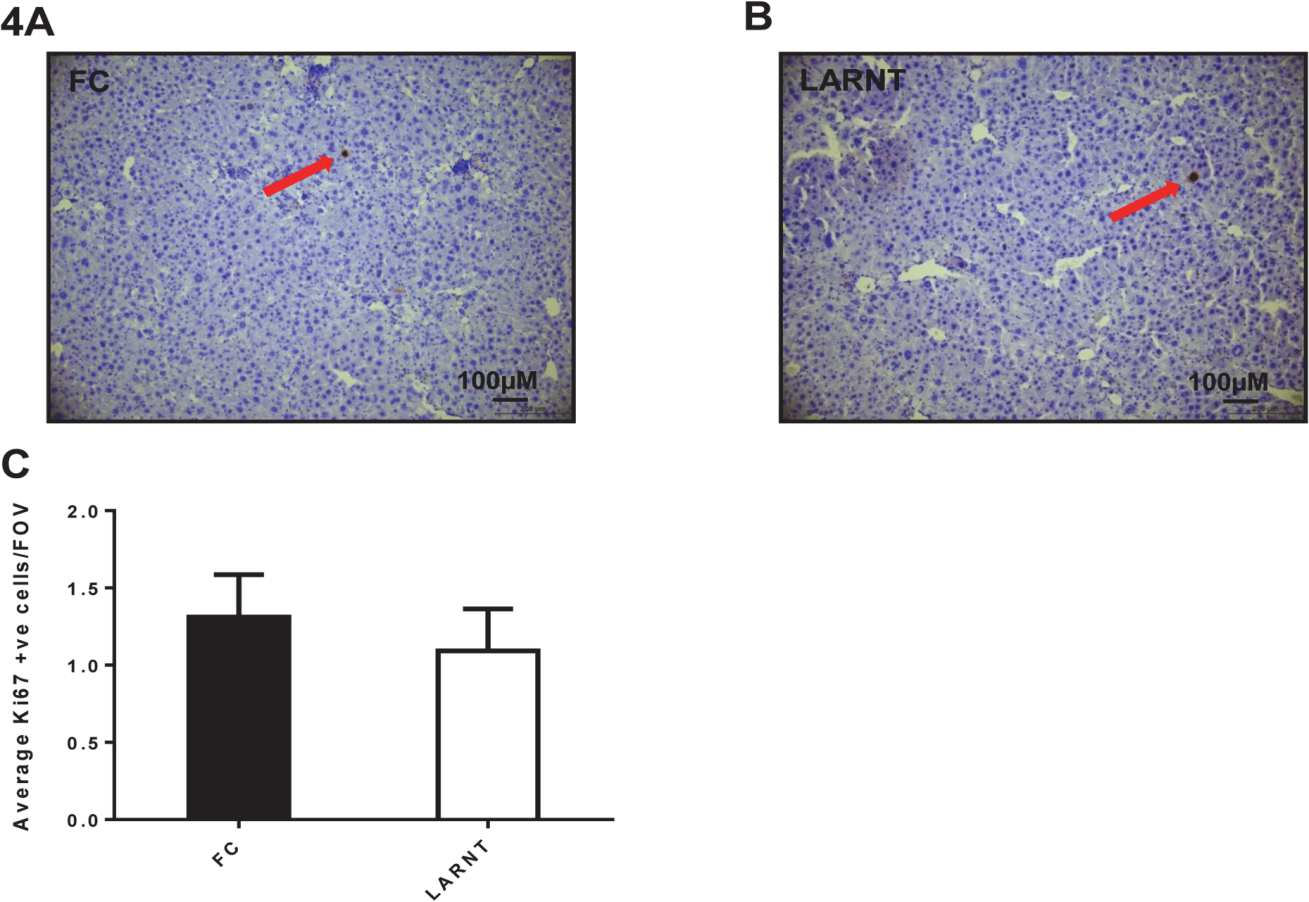
Hepatocyte ARNT deletion does not alter proliferation in the TAA model of fibrosis. Results for FC are shown in black columns and LARNT in white columns. **(A)** Representative histology of FC liver stained with anti-Ki67 at 100X magnification. **(B)** Representative histology of LARNT liver stained with anti-Ki67 at 100X magnification. **(C)** Average Ki67 positive cell counts per field of view at 100 X magnification. Mean±SEM, N = 5-6/group.

Hepatocyte proliferation was assessed by Ki67 staining. There was no significant difference in Ki67 positive cells (**Fig. 4A-C**, p = 0.588).

## Discussion

Hepatocyte *Arnt* deletion overall resulted in similar or lower levels of fibrosis in the TAA induced model of liver injury. LARNT mice had decreased macrophage infiltration and decreased mRNA expression of the pro-fibrotic master regulator *Tgfϐ1*. This occurred alongside a decrease in expression of mRNA for *collagens type 1* and *5*, *Tgfϐ2*, *Timp1* and *Timp2*. Expression of mRNA for apoptotic genes was also altered but did not result in a significant effect on apoptosis. Although there was a decrease in the number of macrophages in LARNT livers after TAA, we found equivalent expression of inflammatory cytokine mRNAs, suggesting similar levels of inflammation. Overall these results are consistent with a role for hepatocyte ARNT in regulation of fibrotic gene expression and macrophage infiltration in response to liver injury.

The results of this study are consistent with previous research which found increased expression of fibrosis genes with increasing liver HIF2-α, and decreased expression with liver HIF1-α deletion [9,17]. However we did not find these alterations affected the pattern of fibrosis in the TAA model. Importantly the aforementioned studies utilised models of ethanol induced liver injury or bile duct ligation, which were not examined in this study.

It is clear that hepatocyte specific deletion of ARNT does not lead to spontaneous fibrosis as occurred in whole body AhR knockout animals [12,13], suggesting perhaps another cell type was responsible for these effects or that AhR deletion perturbed signalling of ARNT and its remaining partners. We found reduced levels of *TGF-ϐ1* and *ϐ2* mRNA suggesting a potential role for ARNT in regulation of transcription. Studies using overexpression or inhibition of TGF-β1 have demonstrated that in the context of liver injury TGF-β1 is pro-fibrotic [20,21]. It is also clear that TGF-β1 also plays a key role in fibrogenesis in humans[22]. TGF-β1 mediates its effects through increasing the expression of connective tissue growth factor (CTGF) and increasing the expression of metalloproteinase inhibitors (TIMPs) and down-regulating the ECM degradative enzymes matrix metallo-proteinases (MMPs) [23,24,25]. In line with decreased TGF-β1 we found decreased mRNA expression of *collagen types 1* and *5* and *Timp 1* and *2*. Importantly previous research has found that hypoxia upregulates *Tgf-ϐ1* and *Col1a1* mRNA expression in dermal fibroblasts, and this data is consistent with these results [26,27]. However, in this model we did not find these alterations in gene expression led to any consistent alteration in liver collagen deposition in the numbers examined.

Previous work has also shown that whole body AhR knockout mice exhibit hepatocyte apoptosis, with additional work suggesting that signalling through AhR may sensitise hepatocytes to FAS induced apoptosis [13,14]. We found alterations in mRNA expression of key apoptosis genes but this did not translate to any significant alterations in Caspase 3 activation as assessed by IHC.

This research shows that although hepatocyte *Arnt* deletion resulted in alterations of profibrotic gene expression and macrophage infiltration in the TAA model of liver fibrosis, overall these alterations resulted in a similar fibrosis pattern. These results suggest that hepatocyte ARNT is not a requirement for initiation of liver fibrosis.
